# The Use of Xpert MTB/RIF Ultra Testing for Early Diagnosis of Tuberculosis: A Retrospective Study from a Single-Center Database

**DOI:** 10.3390/genes14061231

**Published:** 2023-06-07

**Authors:** Cristian Sava, Mihaela Sava, Ana-Maria Drăgan, Alin Iuhas, Larisa Niulaș, Cristian Phillip Marinău, Andreea Bianca Balmoș

**Affiliations:** 1 Faculty of Medicine and Pharmacy, University of Oradea, 410087 Oradea, Romania; 2 Clinical Emergency Bihor County Hospital, 410167 Oradea, Romania

**Keywords:** tuberculosis, molecular testing, Xpert MTB/RIF Ultra, Ziehl–Neelsen staining, Lowenstein–Jensen medium culture

## Abstract

Tuberculosis (TB) is a multisystemic contagious disease produced by *Mycobacterium tuberculosis* complex bacteria (MTBC), with a prevalence of 65:100,000 inhabitants in Romania (six times higher than the European average). The diagnosis usually relies on the detection of MTBC in culture. Although this is a sensitive method of detection and remains the “gold standard”, the results are obtained after several weeks. Nucleic acid amplification tests (NAATs), being a quick and sensitive method, represent progress in the diagnosis of TB. The aim of this study is to assess the assumption that NAAT using Xpert MTB/RIF is an efficient method of TB diagnosis and has the capacity to reduce false-positive results. Pathological samples from 862 patients with TB suspicion were tested using microscopic examination, molecular testing and bacterial culture. The results show that the Xpert MTB/RIF Ultra test has a sensitivity of 95% and a specificity of 96.4% compared with 54.8% sensitivity and 99.5% specificity for Ziehl–Neelsen stain microscopy, and an average of 30 days gained in the diagnosis of TB compared with bacterial culture. The implementation of molecular testing in TB laboratories leads to an important increase in early diagnostics of the disease and the prompter isolation and treatment of infected patients.

## 1. Introduction

Tuberculosis (TB) represents a serious global health issue, and is one of the leading morbidity and mortality factors. Every year, several million people worldwide become infected with tuberculosis and lose their lives due to the disease. TB is a disease linked with poverty and economic stress; vulnerability, marginalization, stigma and discrimination are often problems that people with TB must confront [1].

TB is a multisystemic contagious disease caused by *Mycobacterium tuberculosis* complex bacteria (MTBC). It is estimated that over 1.7 billion people (over 25% of world population) are infected with MTBC. The global incidence had a peak in 2003, and has slowly been decreasing since then. According to the latest World Health Organization (WHO, Geneva, Switzerland) report, the estimated number of deaths from TB experienced a decline between 2005 and 2019, with over 10 million people contracting TB and 1.4 million dying in 2019 and 1.5 million in 2020; however, the estimates for 2020 and 2021 indicate that this trend has been reversed, with an increase in the number of deaths [1,2]. Poverty, HIV infection and drug resistance are the principal factors that contribute to the re-emergence of the global TB epidemic [3]. It is projected that in 2020 and 2021, tuberculosis (TB) will be the second most common cause of death attributed to a single infectious agent, following COVID-19 [1]. About 95% of the cases are recorded in developing countries; one in every nine new cases affects HIV-infected people; and 75% of all cases occur in Africa. It is estimated that 500,000 new multi-drug-resistant TB (MDR-TB) or rifampicin-resistant TB cases occur annually [1].

TB epidemiology varies substantially around the world. The highest prevalence (over 100:100,000 inhabitants) can be observed in Sub-Saharan Africa, India and South-East insular Asia and Micronesia. Intermediary rates (25–99 cases per 100,000 inhabitants) are found in China, Central and South America, Eastern Europe and North Africa. Lower prevalence (under 25 cases per 100,000 inhabitants) can be observed in North America, Western Europe, Japan and Australia [1]. In 2018, there were 52,862 cases reported in the European Union and the European Economic Space (EU/EES), resulting a prevalence of 10.2 cases per 100,000 inhabitants. The prevalence and the incidence in EU/EES countries had declined over the last five years [4]. Unfortunately, Romania remains the country with the highest prevalence from the EU/EES—64.6 cases per 100,000 inhabitants in 2017, which is four times higher than the EU mean, with one of the lowest recovery rates and, at the same time, an annual increase in the infectious reservoir. Romania has a mortality rate due to TB of 4.2 per 100,000 inhabitants, more than six times higher than the EU mean and 1.9 times higher than the WHO European Region’s mean, according to the latest report from the INSP—CNSISP (Romania’s National Public Health Institute, Bucharest, Romania) [5,6].

During the 2014 World Health Summit in Geneva, the WHO proposed a global strategy and targets for tuberculosis prevention, care and control, aiming to stop the global TB epidemic [7]. The proposed objectives were a 95%reduction in TB-related death by 2030, a 90% reduction in disease incidence in the 2015–2035 period and the elimination of associated catastrophic costs for tuberculosis-affected households. In addition to targets for 2030, the End TB Strategy defines 2020 and 2025 milestones for reductions in TB incidence and in the number of TB deaths. The 2020 milestones are a 20% reduction in TB incidence and a 35% reduction in the number of TB deaths, compared with levels in 2015 [8,9]. Reaching these objectives requires the early diagnosis of TB, including through the improvement of diagnostic methods, complete treatment of all people with TB, and the diagnosis and treatment of latent TB infection. The COVID-19 pandemic hugely affected patients’ access to proper medical services. TB care and prevention were particularly affected by the redirection of human, financial and other resources to the COVID-19 response. Furthermore, public health measures resulted in reducing access to TB diagnosis and treatment services [10].

The early diagnosis of tuberculosis enables the prompt initiation of treatment and has the potential to restrict the transmission of this infectious disease. Its diagnosis usually relies on the detection of MTBC in culture. Although this is a sensitive method of detection and remains the “gold standard”, the results are obtained after several weeks. Microscopic examination is an inexpensive and quick test, but is also a rather insensitive test and cannot distinguish between non-tuberculosis mycobacteria and MTBC or between susceptible and resistant strains. Nucleic acid amplification tests (NAATs), being a quick and sensitive method, represent progress in the diagnosis of TB [11]. 

The WHO recommends replacing microscopic examinations, as the initial diagnostic method, with molecular tests capable of identifying MTBC, in certain epidemiological and geographical settings. The newer, more rapid and more sensitive molecular tests recommended for the initial detection of MTBC and drug resistance are designated as mWRDs (molecular WHO-recommended rapid diagnostics tests); these include Xpert MTB/RIF Ultra and Xpert MTB/RIF (Cepheid, Sunnyvale, CA, USA); Truenat MTB, MTB Plus and MTB-RIF Dx tests (Molbio Diagnostics, Goa, India); and loop-mediated isothermal amplification (TB-LAMP; Eiken hemical, Tokyo, Japan) [12].

The Xpert MTB/RIF method is a molecular test that has the capacity to detect the MTBC and the rpoB gene variant associated with rifampicin resistance [13]. Molecular tests are becoming increasingly pertinent in the diagnosis of various diseases as their accessibility and performance capabilities continue to improve [14].

The primary objective of this study was to investigate several hypotheses regarding the efficiency of nucleic acid amplification tests (NAATs) using the Xpert MTB/RIF Ultra method in the early diagnosis of tuberculosis (TB) and its impact on prompt treatment initiation in positive cases. Additionally, the study aimed to assess the ability of this diagnostic approach to reduce false-positive results in suspected TB cases and avoid unnecessary administration of antituberculosis treatment. Moreover, the Xpert MTB/RIF Ultra test was evaluated for its capacity to identify mutations in the rpoB gene associated with rifampicin resistance in samples where *Mycobacterium tuberculosis* complex (MTBC) was detected. The specific objectives of the study were as follows: (i) evaluating the sensitivity and specificity of molecular tests compared to microscopic examination and mycobacterial culture for TB diagnosis, (ii) estimating the time saved in initiating tuberculostatic treatment utilizing molecular tests, (iii) analyzing molecular tests’ ability to identify non-tuberculosis mycobacterial infections and reduce false-positive results, and (iv) detecting rifampicin resistance.

## 2. Materials and Methods

During the period of 1 January 2018–31 December 2020, in the TB Bacteriology Laboratory of the “Dr. Gavril Curteanu” Municipal Clinical Hospital (currently Clinical Emergency Bihor County Hospital) in Oradea, Bihor County, Romania, 13,916 biological specimens were analyzed with the purpose of identifying MTBC. All these samples were tested using microscopic examination and bacterial culture. In 862 cases, the specimens were also tested using the Xpert MTB/RIF Ultra method. 

In this study, 862 patients with a high suspicion of TB infection were included. The suspicion of the disease was determined in accordance with the guidelines provided by the Romanian National Guideline for the prevention, surveillance and control of tuberculosis criteria (epidemiological, clinical and/or imagistic), whose samples were also analyzed using the Xpert MTB/RIF Ultra method, in the mentioned period [15]. The samples consisted of sputum obtained via direct matinal sampling, induced sputum, bronchial aspirate, gastric aspirate, pleural puncture or lumbar puncture (CSF). The quality of the biological samples was essential in obtaining a trustworthy result. Sputum samples deemed inadequate (thin, clear sputum; improper sampling) were excluded from the study.

The collected data were analyzed using IBM SPSS Statistics version 26.

### 2.1. Microscopic Examination Technique

Microscopic examination was performed for all the samples. Sputum was the elective pathological product.

The sputum smear for the microscopic examination was prepared using a bacteriologic wire loop, choosing the spots with purulent, opaque sputum and spreading it on the central portion of the slide, uniformly, in a thin layer, on a surface area of approximately 1 × 2 cm, avoiding the edge of the slide. The slides were left to dry under the hood, at room temperature, and then, heat-fixated using a Bunsen burner (3 times). Ziehl–Neelsen staining was used for acid-fast bacilli (AFB) detection. The slide’s surface was flooded with 0.3% *Fucsina fenica* and heated until steaming. The process was repeated 3–4 times. After 10 min, the slides were rinsed under a gentle flow of water until all free stain was washed away. Decolorization was performed by flooding the slides with 3% acid-alcohol for 3 min, and rinsing them thoroughly with water afterwards. Re-colorization was performed by covering the slides with 0.3% methylene blue for 30 s.

The technique for the other specimens was similar, the only difference being the processing method of the pathological product (prior centrifugation).

After washing and drying the slides, microscopic examination was performed using an optic microscope with an immersion lens (100×) and an ocular lens (10×). The slide was examined over the entire length of the smear. A minimum of 100 fields were examined before the smear was reported as negative (Table 1).

### 2.2. Bacterial Culture Technique

A bacterial culture examination was conducted for all the samples using NaOH method, without centrifugation (dripping method).

From the pathological product, 2–3 mL of purulent particles were extracted using a Pasteur pipette and put into a sterile tube with a threaded cap. An equal amount of 4% NaOH with pH indicator was added. The capped tube was put in a mechanical agitator for 10–15 s. Then, the tube was left at room temperature for 15 min. Neutralization of the sample is performed using 8% HCl until the color turned greenish yellow (neutral pH). The culturing was performed using a single-use pipette.

The used culture medium was Lowenstein–Jensen; for every sample, 3 medium tubes were used. After culturing, the tubes were left in a temperature-controlled room at 37 °C, with the cap half closed, at a 25–30° angle, for 2–5 days. The first reading was taken after 48 h, leaving the tubes vertical afterwards, and eliminating the contaminated tubes. The cultures were monitored weekly until the end of the 8-week period (60 days) of incubation (Table 2).

### 2.3. Xpert MTB/RIF Ultra Test Technique

Xpert MTB/RIF Ultra (Cepheid AB Röntgenvägen 5 171 54, Solna, Sweden) is an automatized molecular test using nested real-time PCR for the qualitative detection of M complex and rifampicin resistance, simultaneously. The primers of this test amplify a region of the rpoB gene containing 81 base-pairs in the core region. The probes are designed to distinguish between wild-type sequences and mutations in the core region, which are associated with rifampicin resistance. The tests were performed using Cepheid GeneXpert^®^ Systems equipment (Cepheid, 904 Caribbean Drive, Sunnyvale, CA, USA), which automatizes and integrates the sample purification, amplifies the nucleic acids and detects the targeted sequence using RT-PCR. 

The system consisted of apparatus, a computer and dedicated software, and it was used for the execution of the test and visualization of the results. The system uses single-use GeneXpert^®^ cartilages which contain the reactive, the RT-PCR process, a sample processing control (SPC) and a probe check control (PCC). Due to the autonomic nature of these cartridges, and the automatic processes, the likelihood of cross-contamination between samples is low. SPC has the role of controlling the bacterial processing and of monitoring the presence of the inhibitor in the PCR reaction. PCC checks the reactive rehydration, the PCR tube feeling, the probe integrity and the colorant stability. Xpert MTB/RIF Ultra simultaneously detects the presence of the *M. tuberculosis* (MTB) complex and rifampicin (RIF) resistance by amplifying the specific sequence form the rpoB gene, which is marked with five signaling molecules (probes A to E) for the mutations of the rifampicin resistance determining region (RRDR). Each signaling molecule was marked with a different fluorophore. The cycle threshold (Ct) was set at 39.0 for the A, B and C probes and at 36.0 for the D and E probes [16].

The Xpert MTB/RIF Ultra test was performed for 536 samples during the duration of the study. For each test, 1 mL of sputum was used, which was sampled with a sterile pipette and transferred into a sealed sterile tube. A total of 2 mL of reactive was added with bactericide and mucus lysis properties. After 10 s of vigorous agitation and 10 min rest at room temperature, followed by further vigorous agitation and 5 min rest, a uniformly homogenized solution was obtained. The content of the tube was transferred to the reaction cartilage using the producer-provided pipette. 

The test took 90 min, and the results were displayed. GeneXpert^®^ Instrument Systems generates results using preestablished algorithms. The interpretation of the measurements is found in Table 3.

## 3. Results

In the observed period a total of 862 patients suspected of TB infection were tested with Xpert MTB/RIF Ultra, Ziehl–Neelsen stain and culture on Lowenstein–Jensen medium. In 2018, 320 (37.1%) tests were performed, in 2019, 289 (33.5%) tests were performed and in 2020, 253 (29.4%) tests were performed. From the study sample, 643 (74.6%) were adults and 219 (25.4%) were pediatric patients.

The collected pathological samples were as follows: 353 (41%)—sputum, 384 (44.5%)—induced sputum, 24 (2.8%)—bronchial aspirate, 73 (8.5%)—gastric aspirate, 7 (0.8%)—pleural fluid, 11 (1.3%)—cerebral spinal fluid and 10 (1.2%)—other pathological products (examples of such fluids include synovial fluid from joints and pus from abscesses located in various regions).

Out of the 862 tested molecular samples, 306 (35.5%) were positive—MTB detected (121 positive samples in 2018, 132 in 2019 and 53 in 2020), and 556 (64.5%) were negative. In the microscopy test, 694 (80.5%) samples were negative and only 168 (19.5%) were positive. Regarding the culture, 299 (34.7%) had a positive culture, 560 (65%) were negative and 3 samples (0.3%) were contaminated (Table 4).

Rifampicin resistance was encountered in 27 cases out of the 306 positive tests (8.82%); in two cases, indeterminate rifampicin resistance was found (0.65%).

**Figure 1:**
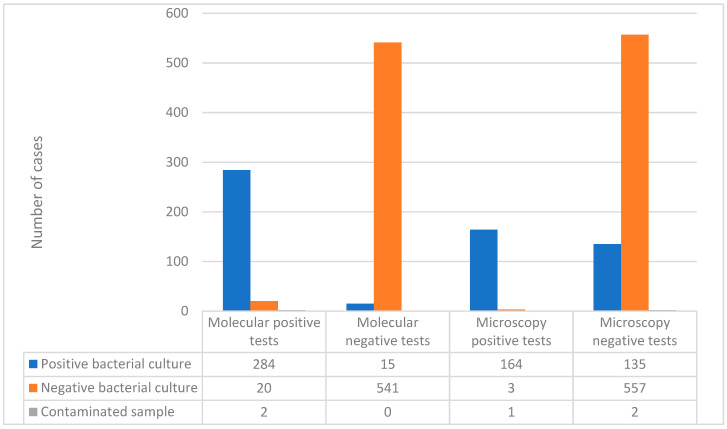
Crosstabulation of molecular and microscopy test results compared with the bacterial culture results.

Out of the 306 patients with detected MTB in the molecular test, 284 (92.81%) had a positive result in the bacterial culture, 20 had a negative culture and 2 samples were contaminated. Of the 556 negative results in the molecular test, 15 had a positive culture. Based on these data, the sensitivity of the Xpert MTB/RIF Ultra test, when compared to the “gold standard” culture, was calculated to be 95%, while the specificity was determined to be 96.4% (Figure 1).

Of the 168 positive result in the Ziehl–Neelsen stain microscopy, 164 (97.6%) had a positive result in the culture, 3 (1.8%) had a negative culture, and 1 (0.6%) sample was contaminated. Of the 694 negative microscopy result, 135 (19.5%) had a positive culture and 2 (0.3%) were contaminated. Based on these data, the microscopy (Ziehl-Neelsen stain) demonstrates a calculated sensitivity of 54.8% and a specificity of 99.5% (Figure 1).

Out of the 168 positive results of the microscopy, 166 had a positive molecular test, and 2 samples were negative. In both cases, the culture was positive for mycobacteria other than tuberculosis (MOTT).

**Figure 2:**
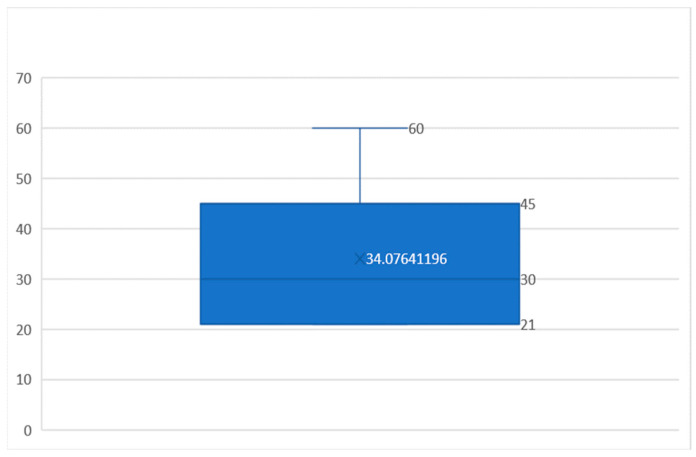
Number of days elapsed from the suspicion of TB until the diagnosis established by the positive culture: indicators of the central tendency (mean: 34.07 days, median: 30 days, min: 21 days, max: 60 days); these values also represent, as all the patients with positive molecular test were immediately started on treatment, the days gained in the early treatment of TB using molecular tests for the diagnosis.

In the 302 cases where the culture was not negative (299 positive samples and 3 contaminated samples), the median time at which the samples were declared positive was 30 days (mean: 34.07 days, min: 21 days, max: 60 days). The majority (135, 44.7%) of the samples were declared positive at the 21-day reading; 53 (17.5%) samples were declared positive after 30 days; 65 (21.5%) were declared positive after 45 days; and 49 (16.2%) samples were declared positive at the 60-day reading. There is a statistically relevant correlation (*p* < 0.0001), inversely related, between the duration of the positive determination and the number of colonies isolated in the culture (Figure 2). 

## 4. Discussion

The early detection and prompt treatment of positive cases are the most effective measures in controlling the spread of tuberculosis [15]. 

The most reliable method of TB diagnostics is bacteriological culture, which is performed, in most cases, using sputum sampled directly, but other pathological products may also be used. The sampling process is essential in ensuring the quality of the result.

The microscopic examination of the pathologic product is extremely relevant in the control of tuberculosis, helping to identify the patients with the highest contagion rate. This method aims to identify AFB in the pathologic product; the test is later confirmed via bacteriological culture. However, microscopic examination using the Ziehl–Neelsen staining technique, although it is a fast, cheap method, has a low sensitivity, and it is not able to distinguish between MTBC and other non-tuberculosis mycobacteria [17]. For AFB to be detected, at least 10^4^ CFU/mL must exist in the pathologic product [18]. Culture confirmation of a TB infection may take 21 to 60 days. Furthermore, neither microscopic examination nor culture can distinguish drug-susceptible TB strains from drug-resistant ones [12].

The testing using nucleic acid amplification tests offered quick and precise diagnosis of tuberculosis, with a sensitivity rate of 95% and a specificity rate of 96.4%. Using this test shortens the isolation period of suspected patients and prevents useless treatment [17,19].

The sputum samples with negative microscopic examination results but with a later positive culture had a lower bacterial load compared with the samples with positive microscopic examination results. With high sensitivity, the NAAT method can detect MTB even in microscopic-negative samples. 

TB patients coinfected with HIV are known to have a low bacterial load compared with the patients without HIV, even though these patients, untreated, have a more aggressive form of the disease [16]. This study cohort did not include any HIV patients. 

The utilization of NAAT was initially approved in 1995 for patients with positive microscopic examination and clinical signs suggestive of TB [19]. The recent progress in molecular testing for MTBC includes the Xpert MTB/RIF Ultra test, which allows for the simultaneous detection of tuberculous bacilli and rifampicin resistance. Patients with negative result following this test can avoid isolation, and those with positive results may benefit from early treatment [20].

The advantages of NAAT include the possibility of early diagnosis and the prompt initiation of treatment, resulting a shorter period of contagion. Moreover, the quick differentiation of patients with MTBC from those infected with non-tuberculosis mycobacteria prevents inadequate and useless treatments and useless investigations of patients’ families [21].

However, there are some limitations to molecular testing interpretations: these methods can have slightly lower sensitivity than bacterial cultures; a negative molecular result does not absolutely exclude the diagnosis of tuberculosis. Furthermore, some sporadic errors in the system may lead to false-positive results in molecular testing [21]. Conventional microscopy and culture remain essential in the evaluation of disease response to treatment [12].

In this study, we reported several situations in which we had a positive molecular test using the Xpert MTB/RIF Ultra method that had a negative microscopic examination and negative culture. We also reported situations with negative molecular testing and negative microscopy but with positive culture.

**Figure 3:**
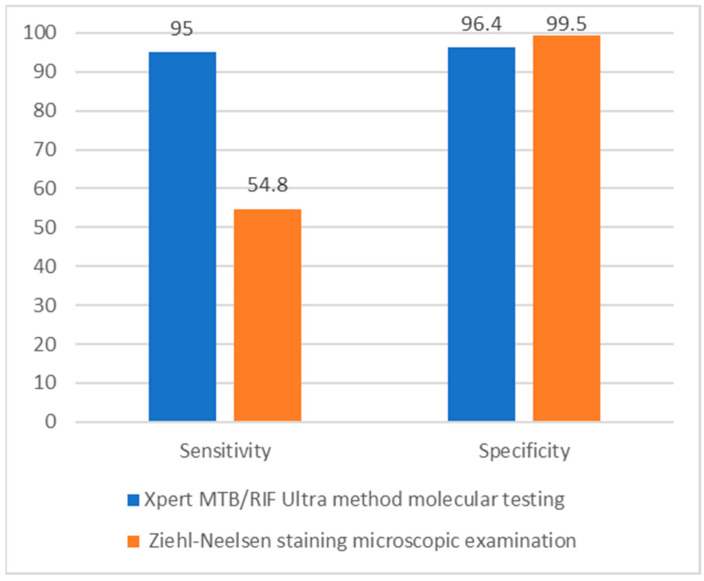
Sensitivity and specificity comparison between molecular testing and microscopic examination in the diagnosis of TB.

As can be seen in Figure 3, molecular testing has superior sensitivity compared with microscopic examination (95% compared with 54.8%). Regarding specificity, the two methods (molecular and microscopic) had similar results (96.4% and 99.5%, respectively). From this, it can be concluded that molecular testing has an important role in the early diagnosis of TB.

In cases in which the Xpert MTB/RIF Ultra test was positive and the initial microscopy was negative, the initialization of the treatment would have been delayed by an average of 34.07 days. In these situations, molecular testing enables the prompt initialization of treatment, which has an impact on the evolution of the disease and the spreading of the disease. Similar results were reported in previous studies, such as Laraque et al. or Luetkemeyer et al. [22,23], but they are slightly different from the CDC reports that cite a 50–80% detection rate in the case of molecular tests performed on negative samples following microscopic examination [24]. 

## 5. Conclusions

The results of the present study validate the recent WHO recommendations; the implementation of molecular testing in TB laboratories leads to important increases in early diagnostics, and has superior sensitivity and similar specificity to microscopic examination.

Molecular testing allows for a quicker diagnosis compared with bacterial culture (90 min vs. weeks), which leads to prompter isolation and treatment of infected patients.

The capacity to distinguish between *M. tuberculosis* and non-tuberculosis mycobacteria shortens the isolation period and prevents the unnecessary treatment of suspected patients. 

Molecular testing can identify rifampicin-resistant strains (and other resistances), allowing for a personalized approach to the treatment of TB patients.

## Figures and Tables

**Table 1:** Semiquantitative expression of the microscopic examination results.

Number of AFB under Ziehl–Neelsen Staining	Result
0 AFB	Negative
1–9 AFB/100 fields	Positive, scanty (exact value)
10–99 AFB/100 fields	Positive 1+
1–10 AFB/field	Positive 2+
>10 AFB/field	Positive 3+

**Table 2:** Semiquantitative expression of the Lowenstein–Jensen solid medium culture results.

Mycobacterium Growth	Result
Absence of colonies	Negative
Under 30 colonies	Positive, scanty (exact value)
30–100 colonies	Positive 1+
Over 100 colonies	Positive 2+
Uncountable conflated colonies	Positive 3+
3 or 2 tubes contaminated and a tube without Bacterial growth	Contaminated

**Table 3:** Possible results of the Xpert MTB/RIF Ultra test.

MTB detected/rifampicin resistance detected
MTB detected/rifampicin resistance not detected
MTB detected/rifampicin resistance indeterminate
MTB not detected
Invalid result

**Table 4:** Distribution of negative and positive results in the three TB tests studied.

Test	Positive	Negative	Total
Xpert MTB/RIF Ultra test	306 (35.5%)	556 (64.5%)	862 (100%)
Ziehl–Neelsen stain microscopy	168 (19.5%)	694 (80.5%)	862 (100%)
Culture on Lowenstein Jensen medium	299 (34.7%)	560 (65%)	859 (99.7%) *

* In 3 cases, the culture was contaminated.

## Data Availability

Not applicable.
